# Minimally Invasive Limited Ligation Endoluminal-Assisted Revision (MILLER): A Review of the Available Literature and Brief Overview of Alternate Therapies in Dialysis Associated Steal Syndrome

**DOI:** 10.3390/jcm7060128

**Published:** 2018-05-29

**Authors:** William W. Sheaffer, Patrick T. Hangge, Anthony H. Chau, Sadeer J. Alzubaidi, M-Grace Knuttinen, Sailendra G. Naidu, Suvranu Ganguli, Rahmi Oklu, Victor J. Davila

**Affiliations:** 1 Department of General Surgery, Mayo Clinic Arizona, Phoenix, AZ 85054, USA; hangge.patrick@mayo.edu; 2 Division of Vascular Interventional Radiology, Minimally Invasive Therapeutics Laboratory, Mayo Clinic, Phoenix, AZ 85054, USA; alzubaidi.sadeer@mayo.edu (S.J.A.); knuttinen.grace@mayo.edu (M.-G.K.); naidu.sailen@mayo.edu (S.G.N.); oklu.rahmi@mayo.edu (R.O.); 3 Division of Vascular Surgery, Mayo Clinic Arizona, Phoenix, AZ 85054, USA; chau.anthony@mayo.edu (A.H.C.); davila.victor@mayo.edu (V.J.D.); 4 Division of Interventional Radiology, Massachusetts General Hospital, Harvard Medical School, Boston, MA 02114, USA; sganguli@partners.org

**Keywords:** MILLER, dialysis associated steal syndrome, high flow, arteriovenous fistula, arteriovenous fistula banding

## Abstract

Dialysis associated steal syndrome (DASS) is a relatively rare but debilitating complication of arteriovenous fistulas. While mild symptoms can be observed, if severe symptoms are left untreated, DASS can result in ulcerations and limb threatening ischemia. High-flow with resultant heart failure is another documented complication following dialysis access procedures. Historically, open surgical procedures have been the mainstay of therapy for both DASS as well as high-flow. These procedures included ligation, open surgical banding, distal revascularization-interval ligation, revascularization using distal inflow, and proximal invasion of arterial inflow. While effective, open surgical procedures and general anesthesia are preferably avoided in this high-risk population. Minimally invasive limited ligation endoluminal-assisted revision (MILLER) offers both a precise as well as a minimally invasive approach to treating both dialysis associated steal syndrome as well as high-flow with resultant heart failure. MILLER is not ideal for all DASS patients, particularly those with low-flow fistulas. We aim to briefly describe the open surgical therapies as well as review both the technical aspects of the MILLER procedure and the available literature.

## 1. Introduction

In 2015, 30 million American adults were known to have chronic kidney disease with approximately 124,000 new cases of end stage renal disease reported [1]. In the absence of transplant, treatment options for end stage renal disease rely heavily on hemodialysis. Currently, 500,000 patients undergo hemodialysis annually and management of their hemodialysis access remains an important aspect of their care [1]. Permanent hemodialysis access options, such as the arteriovenous (AV) fistula and AV graft, are preferred. The Fistula First Initiative was developed to encourage preferential use of arteriovenous fistulas compared to catheters and synthetic grafts as it provides the best long-term patency rates, improves quality of life, and is associated with the lowest mortality rates. However, both autologous AV fistulas and synthetic grafts can have complications, including dialysis access steal syndrome (DASS) and high flow with resultant heart failure [2,3,4].

Dialysis-associated steal syndrome (DASS) is defined as the development of ipsilateral ischemic symptoms in the presence of a functioning fistula or graft [5]. DASS is thought to be the result of three separate etiologies which include arterial stenosis, high fistula flow, and a lack of collateral vasculature and adaptation to the new fistula [2,6]. To diagnose DASS, a patient must have symptoms consistent with steal syndrome (numbness in fingers and hand, cool extremities, tingling, nail changes, sensory and/or motor deficits, pain at rest, tissue loss, and/or ulcerations) lack of distal pulses, as well as radiographic evidence of steal [7]. Findings on angiography consist of a lack of contrast flowing distal to the fistula or contrast taking greater than 10 s to reach the palmar arch [7].

While physiological steal can be present on angiography in as many as 90% of arteriovenous fistulas, DASS is only observed in approximately 1–8% of AV fistulas and grafts created [2,8]. If symptoms are mild, close monitoring and observation for symptom stability or improvement is reasonable [9,10]. Symptoms such as rest pain or digital ischemia mandate intervention. At present there is a variety of treatment options for DASS with the most definitive being fistula ligation [2,9]. Ligation of the AV fistula offers resolution of DASS, however patients invariably require alternative access. The ideal management of DASS offers improvement of symptoms without loss of dialysis access. Other modalities for management of steal syndrome include banding, plication, distal revascularization-interval ligation (DRIL), revision using distal inflow (RUDI), and proximalization of arterial inflow (PAI). Recently, the minimally invasive limited ligation endovascular revision (MILLER) procedure has garnered interest for its minimally invasive approach and measured diameter banding.

This overview will serve to review the current approaches to surgical management of DASS. More specifically, we aim to summarize the MILLER procedure from a technical standpoint and review the pertinent literature.

## 2. Surgical Management

### 2.1. Distal Revascularization-Interval Ligation (DRIL)

**Figure 1:**
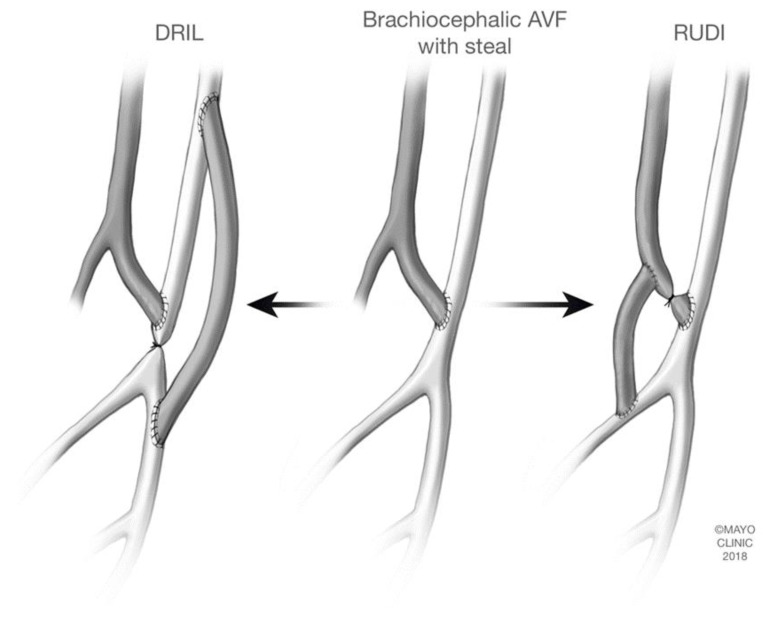
Revascularization for steal syndrome of a brachiocephalic arteriovenous fistula (AVF). Brachiocephalic AVF is shown in the center. The darker vessel represents the vein and the lighter vessel represents the artery. DRIL, distal revascularization-interval ligation, is shown on the left. The native artery is ligated distal to the AV anastomosis. Bypass is performed from the native artery proximal to the vascular access take off, to the native artery distal to the site of ligation. The bypass vessel is depicted in the dark color as a saphenous vein graft is commonly used. RUDI, revascularization using distal inflow, is depicted on the right. The original fistula is ligated and the AV anastomosis is relocated distally onto a smaller artery to limit flow to the AVF and also to increase flow through the ulnar artery. Copyright 2018 Mayo Foundation for Medical Education and Research.

The DRIL procedure is a well-established surgical option for DASS which involves ligation of the native artery just distal to the anastomosis with subsequent bypass from the native artery, proximal to the vascular access takeoff, to the native artery distal to the site of ligation (Figure 1) [11]. The proximal anastomosis of the bypass is preferably performed at least 5–7 cm proximal to the vascular access takeoff in an end-to-side fashion [11,12,13]. A saphenous vein graft is the most commonly used conduit however synthetic grafts may be used.

Early reports of DRIL success released in the mid-1990s were very promising and suggested successful resolution of symptoms in nearly 100% of patients and access patency rates of 84% [11]. Recent studies demonstrate symptomatic relief is seen in 83–100% of patients undergoing DRIL for steal syndrome while maintaining secondary access patency in 73–98% of patients [7,9,10]. Complications range from 5–17% and largely consist of surgical site infections and access thrombosis [7,10].

Frequently cited concerns with DRIL as initial therapy for steal syndrome include the use of general anesthesia in a high risk patient population as well as ligation of the brachial artery for the maintenance of a known temporary access. Despite these concerns, DRIL remains a well-studied and frequently used treatment strategy in the patient with steal syndrome.

### 2.2. Revascularization Using Distal Inflow (RUDI)

First described in 2005, RUDI aims to decrease the radius of the access while maintaining forward flow in the native artery. The primary theoretical benefit of RUDI over DRIL is that RUDI will maintain native circulation, as opposed to ligation of the brachial artery frequently performed in the DRIL procedure [14,15]. If graft failure occurs, this would result in loss of access rather than limb ischemia [14]. The original fistula outflow tract is ligated. Two new anastomoses are created. The AV anastomosis is relocated distally along the artery. A smaller artery is used, frequently the radial artery, to limit flow through the AV access while increasing flow through the ulnar artery. The fistula is reconstructed by anastomosing the graft to the original venous outflow tract (Figure 1). To successfully perform RUDI, patients must have a minimum radial or ulnar target of 2 mm, an intact second artery to the hand, and minimal calcifications [15].

In Minion’s initial case series, four patients had successful improvement in symptoms following the initial procedure. Additional series by Callaghan yielded an impressive 100% improvement of symptoms however there was loss of access in three of seven patients in the series [16]. Loh et al. have the largest and most recent series of 29 RUDI procedures which has shown improvement in symptoms in 100 percent of steal patients (79% complete, 21% partial) with primary and secondary patency rates of 74% and 87% [15]. The RUDI procedure remains a less commonly performed, however reasonable alternative to the DRIL without the concerns of brachial artery ligation.

### 2.3. Proximalization of Arterial Inflow (PAI)

**Figure 2:**
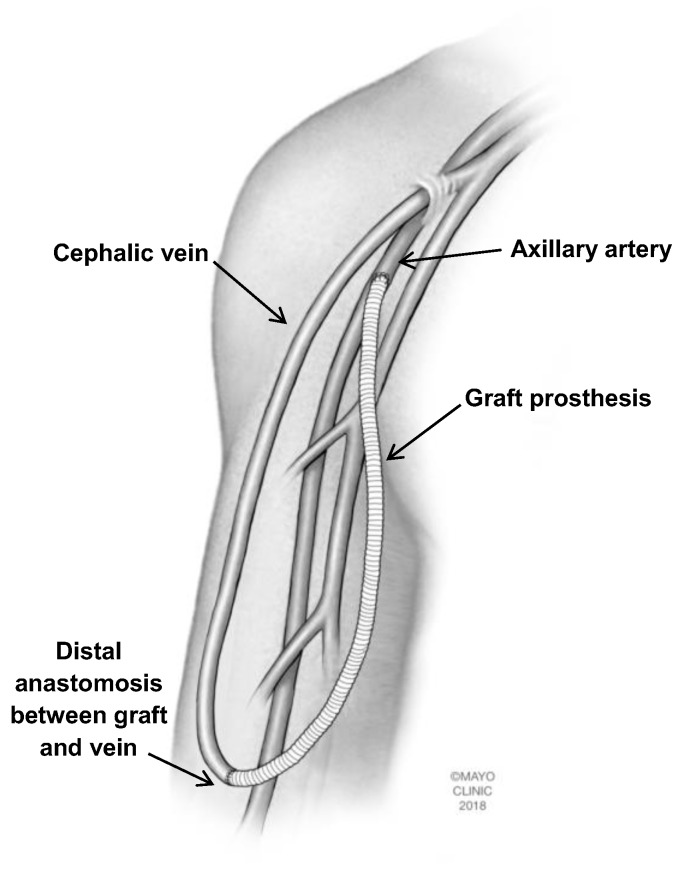
Proximalization of arterial inflow (PAI). The existing arteriovenous fistula is dissected and venous outflow is ligated (not shown). A small diameter graft prosthesis is used to deliver proximalized arterial inflow to the new access site. The proximal end of the graft is anastomosed in a side-to-end fashion to the artery and the distal end is anastomosed in an end-to-end fashion to the vein. Copyright 2018 Mayo Foundation for Medical Education and Research.

PAI was first described in 2006 as a procedure primarily for patients with low flow steal syndrome (<800 mL/min for native fistulas and <1000 mL/min in prosthetic access) [17]. PAI has similar efficacy to DRIL, but avoids brachial artery ligation. The procedure is based on using a small diameter graft to proximalize the arterial inflow of the access which should yield a decreased pressure drop distal to the anastomosis while maintaining similar access flow [17]. The procedure involves dissection of the existing anastomosis and proximal artery with ligation of the venous outflow. A synthetic graft is tunneled subcutaneously with the arterial anastomosis performed in a side-to-end fashion and the venous anastomosis being performed end-to-end (Figure 2). Zanow et al. also described the use of intraoperative arterial pressure monitoring to document improved distal flow.

Zanow’s initial series included 30 patients, 84% experienced complete symptom resolution and 16% experienced symptom improvement. Mean access flows were quantified and did not decrease significantly from pre-operative flows. Twelve-month primary and secondary patency were excellent at 87% and 90% respectively. Secondary patency was 78% at 36 months [17].

In two further studies by Thermann et al., PAI was reviewed in 23 and 36 patients respectively [18,19]. In their early series, complete resolution of symptoms was achieved in 65% of patients and improvement seen in 26% with secondary patency of 85% at 18 months. In the subsequent series, 40 PAIs were performed and clinical success was realized in 90% with 82% having permanent success. Primary patency was 62% at one year and secondary patency was 75% at 18 months [18,19].

### 2.4. Open Surgical Banding and Plication

**Figure 3:**
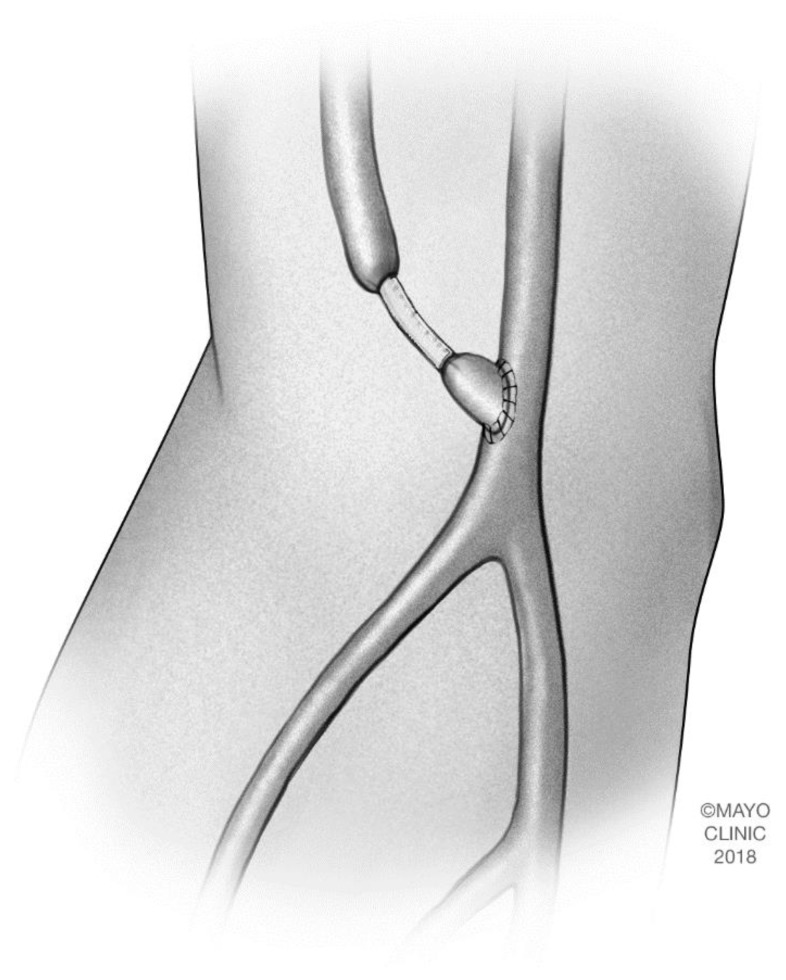
Banding of a brachiocephalic arteriovenous fistula. The band is placed around the outflow tract of the fistula to create a narrowing which increases resistance to flow and therefore, peripheral perfusion. Copyright 2018 Mayo Foundation for Medical Education and Research.

Banding and plication are both procedures which rely on increasing the resistance to flow, thereby increasing peripheral perfusion [20,21]. Banding is frequently performed by wrapping a segment of polytetrafluoroethylene (PTFE) around the outflow track of the fistula to create a narrowing. (Figure 3). Proponents of surgical banding argue that this requires minimal intervention and maintenance of the dialysis access. However, it lacks an accurate way to determine the degree of narrowing that will address symptoms [5,7]. Digital pulse oximetry can help in identifying the amount of compression which will yield symptom improvement, but defined criteria guiding the degree of compression are lacking. Previous studies evaluating the banding technique have realized unacceptable thrombosis rates of 19–90% [9,21,22,23]. Banding is usually reserved for patients with high flow AV accesses. At present, open banding techniques are not commonly reported. 

Fistula plication involves suture guided narrowing of a short segment of the proximal venous outflow tract. The degree of narrowing is guided by return of palpable pulses or dopplerable signals. Recent limited series have shown promise with success rates of 92–100% with revision rates of 14–20% [21,24]. Further, more robust, series are needed before this procedure becomes widely adopted.

## 3. MILLER Procedure

**Figure 4:**
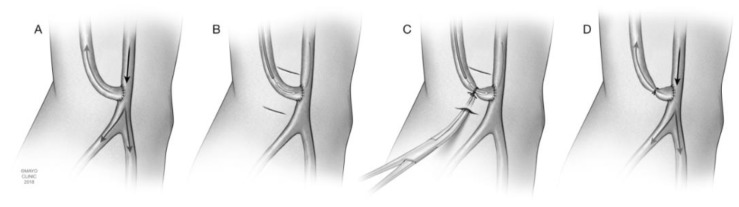
MILLER (minimally invasive limited ligation endoluminal-assisted revision) steps. (**A**) A majority of blood flow is diverted into the brachiocephalic arteriovenous fistula (AVF), resulting in less flow to the distal extremity, resulting in steal syndrome. (**B**) Angioplasty balloon traversing the AVF anastomosis in a retrograde fashion. Incisions are made cranially and caudally to the fistula. (**C**) Dissection is performed with tunneling of a hemostat beneath the fistula. Monofilament suture is also passed underneath the fistula. The suture is tied firmly around the fistula, but still below the skin, with the endovascular balloon in place as a guide. (**D**) Post-procedural blood flow is redirected with more blood flowing distally. Copyright 2018 Mayo Foundation for Medical Education and Research.

The minimally invasive limited ligation endoluminal-assisted revision, or MILLER, procedure offers a standardized method to traditional banding procedures for treating DASS (Figure 4).

MILLER is initiated by accessing the fistula along the outflow tract in a retrograde fashion. A 0.035 glide wire is used with a 4 French catheter to traverse the arterial anastomosis. Arteriography is performed to rule out both proximal inflow lesions and outflow obstruction (Figure 4B). Arterial inflow stenoses are treated endovascularly. After assurance of adequate arterial inflow, the diameters of arterial inflow (usually the brachial artery), distal arteries (radial and ulnar), and banding site are determined from angiography to assist in determining the size of angioplasty balloon to utilize [5,7]. For patients with steal, the size of the initial balloon chosen is smaller in diameter than the distal arteries, most frequently 3–5 mm. For patients with high flow AV access, the nomogram by Murray et al. is used with a typical goal of 60–80% reduction in diameter of the outflow [20].

**Figure 5:**
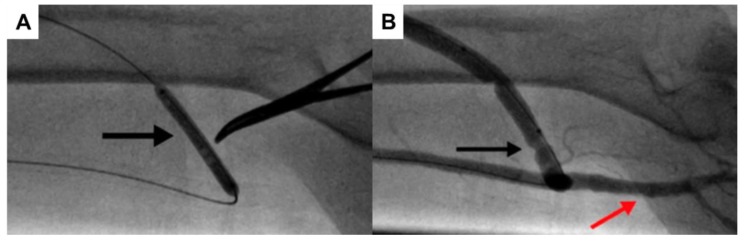
Angiography during minimally invasive limited ligated endoluminal-assisted revascularization (MILLER) procedure. (**A**) An inflated 5 mm angioplasty balloon (black arrow) is guided to the proximal arteriovenous graft (AVG) site. A hemostat is used to designate the area for skin incisions. (**B**) Post-procedure imaging demonstrates a waist in the AVG (black arrow) and distal flow in the brachial artery (red arrow).

Attention is then directed to the patient’s arm where the fistula and anastomosis are identified by palpation and confirmed with angiography. Within approximately 1–3 cm of the anastomosis, incisions are made on either side (cranially and caudally) to allow circumferential control of the fistula. A 2-0 monofilament suture is passed around the fistula in the plane of dissection (Figure 4C). The angioplasty balloon is placed endovascularly at the location of the dissection. The 2-0 suture is then tied firmly around fistula with the balloon in place intravascularly as a guide (Figure 4D). A waist can be seen on imaging after the suture is tied (Figure 5).

**Figure 6:**
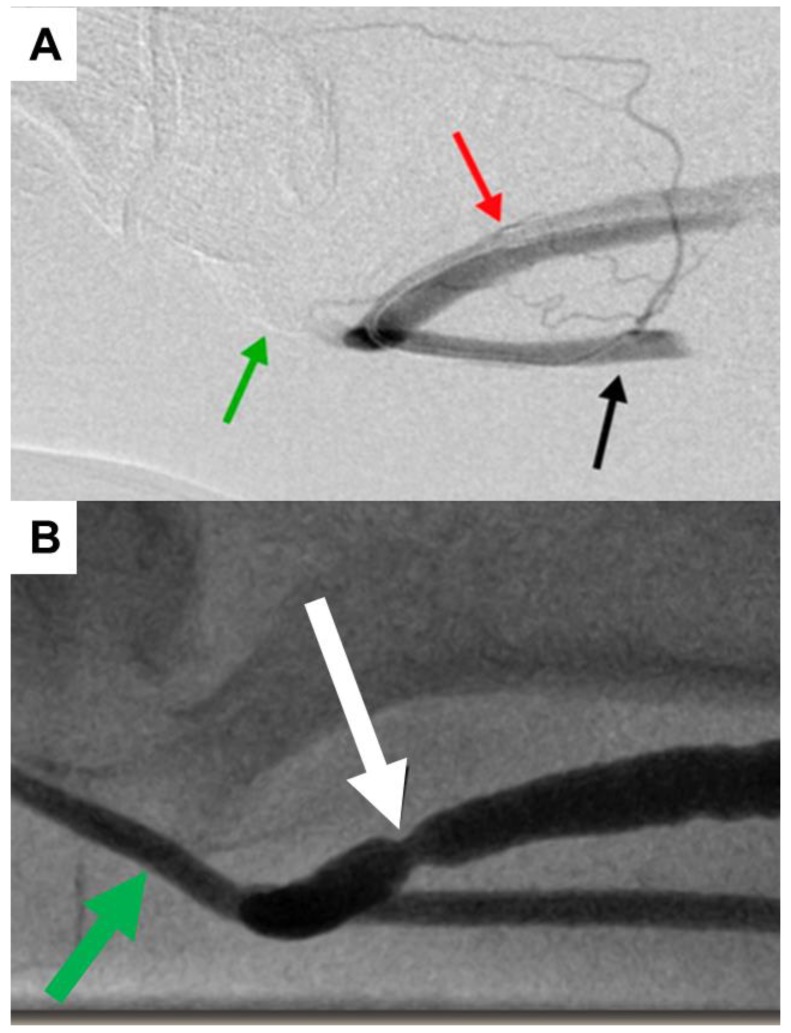
Pre- and post-procedural angiographic images of an arteriovenous graft (AVG) following minimally invasive limited ligation endoluminal-assisted revision. (**A**) Pre-procedural imaging following brachial artery injection (black arrow) immediately proximal to the anastomosis of the AVG. Contrast is completely diverted into the AVG (red arrow), while no flow is seen distally into the artery (green arrow). (**B**) Post-procedural imaging shows a newly formed waist (white arrow) in the proximal AVG after the suture has been tied with redistribution of blood flow distally into the brachial artery (green arrow).

After the suture is secured, the balloon is deflated and removed. The patient is evaluated for both subjective and angiographic resolution of steal, i.e., reversal of flow in the distal arteries or sluggish distal perfusion. As noted in Figure 6A, preoperative angiography from the brachial artery (black arrow) shows no flow distally past the graft (green arrow). Post-procedural imaging in Figure 6B demonstrates redistribution of flow distally (green arrow) due to narrowing of the outflow (white arrow).

If there is no improvement in symptoms or persistent angiographic evidence of steal, an additional band may be placed approximately 0.5 cm distal to the initial band to increase the length of the high resistance segment. If access flow is limited, angioplasty can be utilized to dilate the band [5,7].

Complications overall are quite low in the MILLER procedure. Intra-procedure bleeding was the most common reason for difficulties or abandonment of the procedure itself in previous series. A recent case report describing the development of a 2 cm pseudoaneurysm as a result of injury to the access during the dissection highlights the potential pitfalls of a partially blind circumferential dissection and the importance of intraprocedure identification of injuries to the dialysis access [25].

The MILLER procedure was first reported by Dr. Goel and Dr. Miller in a series of 16 patients. The procedure was determined to be successful in all 16 cases as defined by improvement in symptoms and at least one successful dialysis run. Four-mm bands were most commonly used (14/16 patients) with two 5-mm bands used on the remaining patients. The authors reasoned that a 3-mm band would cause too much restriction and likely thrombosis however a 6-mm band would be ineffective in redirecting the flow to the distal forearm arteries. The mean follow-up for the study was limited to three months. Secondary patency in this cohort was later reported at 77% at 36 months. There were no infections or other complications reported in this series [5]. In 2010, Miller published a review of 183 patients undergoing the MILLER procedure [7]. This study included both patients with true steal (*n* = 114) and those with signs/symptoms of high flow (*n* = 69). Among all patients, 156 were upper arm accesses and 27 were forearm accesses. The most common band size was 4 mm for fistulas and 3 mm for grafts. The final 20 procedures in this series were performed with the assistance of flow and pressure measurements. Average reduction of flow was 50% for steal patients and 52% for high-flow patients. In this series, 109 of 114 (96%) steal patients and 69 of 69 (100%) high flow patients had complete symptom relief. Of patients with complete symptom relief, 89% of steal patients and 94% of high flow patients achieved clinical success with a single banding procedure. Patients who required multiple bandings frequently had a comorbidity of hypertension and occlusion of one forearm artery with incomplete palmar arch on angiography. High-flow patients requiring multiple bandings all had proximal brachial artery hypertrophy. Mean follow-up for this study was 11 months with a range of approximately 1 week to 37 months. Primary band patency for high-flow patients was 75 and 85% at six months. Primary access patency for steal and high flow patients was 52% and 63% at three months. Secondary patency was 90% at 24 months. The procedure was successfully performed in 225/229 (98%) attempts with access site bleeding being the limiting factor in the four abandoned procedures (Table 1). Major complications included three patients with cellulitis requiring graft removal within two weeks of the original procedure. Additionally, three patients developed post procedure bleeding which was managed with manual compression. No patients required open surgical revision.

Subsequent studies have shown the MILLER procedure to provide variable success in patients with steal. Shukla et al. performed a retrospective review of 20 patients undergoing 30 MILLER procedures. The goal luminal reduction was 75% and the most commonly utilized band size was 2.5 mm (range 2.0–3.5 mm). No pressure or flow monitoring was utilized in this series. The authors defined technical success as preserved flow on completion angiography and this was achieved in all 30 procedures. Clinical success, defined as relief of symptoms, was achieved in 75% of patients after one banding procedure and 95% of patients after two procedures. Seven patients underwent one repeat procedure and one patient underwent three repeat procedures. MILLER band patency was 83% at one month and 77% at six months. Post intervention assisted primary patency was 95, 93, and 92% at 3 months, 6 months, and 12 months (Table 1) [26]. Mean follow-up was 552 days (range 5–1644 days).

Shintaku et al. performed a smaller retrospective review with some modifications to the original described MILLER procedure [27]. These included the use of flow volume monitoring of the brachial artery using Doppler ultrasonography and the use of a finger pulse oximeter in patients with steal. In the series, seven patients underwent the procedure for high-flow and five patients for DASS. Outcomes in the study focused primarily on decrease in flow and improvement in digital pulse oximetry rather than symptom resolution. In the high-flow group, a mean flow decrease from 2043 mL/min to 1248 mL/min was noted, resulting in a mean reduction of flow by 39%. Band sizes in this group ranged from 3–6 mm, with 3.5 mm most commonly used. In the steal group, balloon sizes were smaller, ranging from 2–4 mm, therefore resulting in a greater reduction in diameter post-procedure. There was reported improvement in all patients’ pulse oximetry readings however the degree of improvement was not presented. Despite this reported improvement in pulse oximetry reading, the authors admit there was not complete symptom resolution in any of the five steal patients. The mean flow reduction for the steal group was from 997 mL/min to 548 mL/min. Only secondary patency rates were reported in this series. For the high-flow group secondary patency was 83% both at 6 and 12 months. In the steal group secondary patency was 50% at six months and 25% one year. Ultimately, this limited series provided quantifiable decreases in access flow in high flow patients, however clinical success in the steal group was limited with an unacceptably high failure rate.

## 4. Conclusions

Given the promising data from limited studies, the MILLER procedure appears to be a reasonable first line therapy for any patient with a high flow fistula and most patients exhibiting signs/symptoms of steal. Compared to traditional banding, MILLER offers a minimally invasive approach for creating a precise stenosis to produce the desired hemodynamic change. It is best suited for patients with steal syndrome and high flow fistulas, as reduction in flow of low flow fistulas could result in access thrombosis. As evidenced in the most recent MILLER series, there does appear to be a learning curve and outcomes seem to be associated with number of procedures being performed in a given practice. The procedure also requires angiography capabilities which may not be available in every center. Additional larger studies evaluating the utilization of intra procedure flow and pressure monitoring, as Miller et al. did in their final 20 procedures, may contribute to further increasing the precision of the procedure and possibly improved rates of symptom improvement and postprocedure patency.

## Figures and Tables

**Table 1:** Comparison of available MILLER procedure series

Group	Presentation	No. Patients	Clinical Success		Band Patency	Primary Access Patency	Secondary Access Patency	Follow Up (Months)
			One Banding	Multiple Bandings				
Goel et al. [5]	Steal	16	N/A	N/A	100% ^1^	100% ^1^	77%	3, 36
Miller et al. [7]	HF	69	94%	100%	85% ^2^	63% ^1^	89% ^4^	11
	Steal	114	89%	96%	75% ^2^	52% ^1^	90% ^4^	
Shukla et al. [26]	Steal	20	75%	95%	77% ^2^	95% ^1,^*	86% ^1,‡^	18
Shintaku et al. [27]	HF	7	N/A	N/A	N/A	N/A	83% ^3^	46
	Steal	5	0%	0%	N/A	N/A	25% ^3^	

HF = High-Flow; N/A = not available; ^1^ 3 months; ^2^ 6 months; ^3^ 12 months; ^4^ 24 months. * Post intervention assisted primary patency. ^‡^ Low follow-up limited long term secondary patency data.
